# Afucosylated IgG Promote Thrombosis in Mouse Injected with SARS-CoV-2 Spike Expressing Megakaryocytes

**DOI:** 10.3390/ijms26147002

**Published:** 2025-07-21

**Authors:** Meryem Mabrouk, Farah Atifi, Hicham Wahnou, Afaf Allaoui, Nabil Zaid, Abdallah Naya, Ejaife O. Agbani, Loubna Khalki, Meriem Khyatti, Youssef Tijani, Khadija Akarid, Damien Arnoult, Haissam Abou-Saleh, Othman El Faqer, Salma Labied, Mounia Ammara, Fadila Guessous, Farid Jalali, Younes Zaid

**Affiliations:** 1 Materials, Nanotechnologies and Environment Laboratory, Department of Biology, Faculty of Sciences, Mohammed V University in Rabat, Rabat 10100, Morocco; meryemmabrouk4@gmail.com (M.M.); farahatifi97@gmail.com (F.A.); afaf.allaoui@gmail.com (A.A.); salma.labied@um5r.ac.ma (S.L.); 2 Laboratory of Integrative Biology, Department of Biology, Ain Chock Faculty of Sciences, Hassan II University, Casablanca 20200, Morocco; hwwahnou@gmail.com (H.W.); anaya@mabiotech.com (A.N.); mr.othman.elfaqer@gmail.com (O.E.F.); ammara.mounia18@gmail.com (M.A.); 3 Health and Environment Laboratory, Higher Institute of Nursing Professions and Health Technology of Rabat, Rabat 10000, Morocco; zaidnabil@hotmail.com; 4 Department of Physiology & Pharmacology, University of Calgary, Calgary, AB T2N 1N4, Canada; ejaife.agbani@ucalgary.ca; 5 Interdisciplinary Laboratory of Biotechnology and Health, Neurosciences and Cellular Physiology Team, Mohammed VI Higher Institute of Biosciences and Biotechnology, Casablanca 20190, Morocco; lkhalki@um6ss.ma; 6 Department of Biology, Mohammed VI University of Sciences and Health (UM6SS), Casablanca 20180, Morocco; 7 Laboratory of Viral Oncology, Institut Pasteur du Maroc, Casablanca 20360, Morocco; meriem.khyatti@pasteur.ma; 8 Faculty of Medicine, Mohammed VI University of Sciences and Health, Casablanca 20180, Morocco; y.tijani@hck.ma; 9 Biochemistry, Biotechnology and Immunophysiopathology Research Team, Health and Environment Laboratory, Ain Chock Faculty of Sciences, Hassan II University of Casablanca, Casablanca 20200, Morocco; kakarid@yahoo.fr; 10 INSERM, UMR_S 1197, Université Paris-Saclay, 94800 Villejuif, France; damien.arnoult@inserm.fr; 11 Department of Biomedical Sciences, College of Health Sciences, QU Health, Qatar University, Doha 2713, Qatar; hasaleh@qu.edu.qa; 12 Oncopathology, Biology and Environment of Cancer Laboratory, Mohammed VI Center for Research and Innovation, Rabat 10112, Morocco; fguessous@um6ss.ma; 13 Department of Microbiology, Immunology and Cancer Biology, School of Medicine, University of Virginia, Charlottesville, VA 22903, USA; 14 Department of Gastroenterology, Saddleback Medical Group, Laguna Hills, CA 92653, USA

**Keywords:** COVID-19, SARS-CoV-2, afucosylated IgG, megakaryocytes, spike S, platelet activation, thrombosis

## Abstract

Despite the prevalence of fucosylated IgG in plasma, specific IgGs with low core fucosylation sporadically emerge in response to virus infections and blood cell alloantigens. This low fucosylation of IgG is implicated in the pathogenesis of SARS-CoV-2 and dengue infections. In COVID-19, the presence of IgGs with low core fucosylation (afucosylated IgGs) targeting spike protein predicts disease progression to a severe form and actively mediates this progression. This study reveals that SARS-CoV-2 infection of megakaryocytes promotes the generation of pathogenic afucosylated anti-spike IgGs, leading to outcomes, such as pulmonary vascular thrombosis, acute lung injury, and mortality in FcγRIIa-transgenic mice. Platelets from mice injected with virus-infected human megakaryocytes express significant activation biomarkers, indicating a direct link between the immune response and platelet activation. Mice injected with virus-infected human megakaryocytes demonstrate an elevated rate of thrombus formation induced by FeCl_3_ (4%) and a reduction in bleeding time, emphasizing the intricate interplay of viral infection, immune response, and hemostatic complications. Treatment with inhibitors targeting FcγRIIa, serotonin, or complement anaphylatoxins of mice injected with spike-expressing MKs successfully prevents observed platelet activation, thrombus formation, and bleeding abnormalities, offering potential therapeutic strategies for managing severe outcomes associated with afucosylated IgGs in COVID-19 and related disorders.

## 1. Introduction

Coronavirus SARS-CoV-2 is the causative agent of the global coronavirus 2019 (COVID-19) pandemic, which has caused a severe threat to public health since its inception. SARS-CoV-2 infection causes a mild acute disease in the majority of those infected but progresses to severe and fatal disease in a subset [1]. Extensive work has been conducted to elucidate the precise events responsible for disease progression in COVID-19. Two specific early events that accurately predict disease progression have been independently identified. These events include (1) the early interaction between SARS-CoV-2 and megakaryocytes (MKs) in the bone marrow [2,3], and (2) the early production of a distinct form of non-neutralizing antibody against SARS-CoV-2 spike protein that drives the immunopathology of progressive disease [4,5,6].

Multiple reports have shown that the quality of antibodies produced early on in response to SARS-CoV-2 spike protein differs between individuals, and the production of a distinct form of anti-spike IgG early in the illness accurately predicts disease progression [4,5,6]. Specifically, the glycosylation pattern of the Fc component of IgGs formed against the spike protein was shown to determine the fate of outpatients studied in two prospective cohorts [4,6,7,8]. Individuals who, due to unclear factors, produced IgGs with low core fucosylation (afucosyalted IgGs) against the spike protein early in the illness as outpatients consistently progressed to hospitalization and severe COVID-19 [4,6,7]. Those who instead formed anti-spike IgGs with normal levels of core fucosylation mounted an appropriate immune response to SARS-CoV-2 early on, experienced only a mild course and did not progress to severe COVID-19 [4,5,6].

Mechanistically, low core fucosylation of anti-spike IgGs in COVID-19 is known to trigger a potent pro-inflammatory cascade of cytokine production and lung immunopathology driven by intense FcγR-mediated stimulation of macrophages, monocytes, natural killer cells, and platelets [5,6]. Intriguingly, in individuals who produced afucosylated IgG against the spike protein (who subsequently progressed to severe disease), the low core fucosylation was limited to IgG responses against the spike protein, which is expressed on the surface, whereas the pattern of core fucosylation remained largely intact at normal levels in IgGs formed against the viral nucleocapsid protein, which lacks surface expression [4]. This is in alignment with other instances of afucosylated IgG formation in other disorders, where afucosylated IgG production is seen in response to only surface proteins of enveloped viruses and surface alloantigens of host blood cells, such as platelets [2,4,7]. Therefore, the specific surface expression of the spike protein on the viral envelope and on the membrane of infected host cells is hypothesized to play a key role in inducing a pattern of low core fucosylation in certain individuals [7]. However, spike protein is found abundantly expressed on the membrane of various host cells in the respiratory tract during SARS-CoV-2 infection, raising the question of whether specific cell populations expressing spike protein may be required to induce such aberrant afucosylated responses that drive disease progression.

The body of knowledge in regard to afucosylated responses is currently limited. In Fetal and Neonatal AlloImmune Thrombocytopenia (FNAIT), afucosylated responses occur in reaction to alloantigens expressed on the platelets’ membrane and drive the immunopathology in this disorder [8,9]. A hypothesis emanating from this phenomenon would suggest that the expression of spike protein on the membrane of platelets in COVID-19 may similarly induce formation of afucosylated anti-spike IgGs and drive the immunopathology of severe illness. While it is known that whole and infectious SARS-CoV-2 is found within platelets in severe COVID-19, multiple experiments using electron microscopy and immunogold labeling for the spike protein have shown the spike protein to be limited only to the cytoplasmic lumen, and not expressed on the membrane of the infected platelets [3,4]. Bone marrow megakaryocytes (MKs) are, however, known to be infected by SARS-CoV-2 as well, and are believed to be the most likely source of infected platelets in COVID-19 [3,4]. Infected MKs were shown to transfer viral antigens, including the spike protein to emerging platelets during thrombopoiesis [2]. Moreover, infected MKs found early in the illness were predictive of disease progression to severe COVID-19, similar to the predictive nature of early afucosylated anti-spike IgGs [2,3]. Thus far, however, no links between these two predictive factors have been discovered.

On a different note, human platelets express FcγRIIa receptor for IgG-containing immune complexes [10]. Functionally, platelet FcγRIIa binds IgG immune complexes, triggering platelet activation and granule release, thus facilitating clearance of the pathogen, and supporting the immune system [11,12,13]. However, in response to overstimulation of platelet FcγRIIa, platelets can become hyperactive, leading to dysregulated hemostasis, thrombosis, and other complications [11,12]. Building on this, binding of afucosylated anti-spike IgG with high affinity to platelet FcγRIIa has been reported as a plausible mechanism for platelet hyperreactivity and thrombosis in patients with severe COVID-19 [13], whereas factors underlying the formation of these pathogenic anti-spike afucosylated IgGs during SARS-CoV-2 infection remain poorly understood.

Based on the above lines of evidence, a hypothesis was made that, although the spike protein is not found expressed on the membrane of platelets, spike expression on the membrane of platelet progenitors may occur during thrombopoiesis as infected MKs transfer viral material, including the spike protein to budding pro-platelets. Spike expression on the surface of pro-platelets during thrombopoiesis may then trigger the formation of afucosylated IgG against the spike protein, similar to the afucosylated responses seen in reaction to platelet surface alloantigens encountered during/in the FNAIT disorder [8,9]. The resulting afucosylated anti-spike IgGs may then drive the immunopathology of severe COVID-19 by inducing platelet hyperactivation and thrombus formation, thus contributing to thrombotic complications.

In this study, we demonstrated that SARS-CoV-2-infected human MKs may serve as a crucial element that triggers the formation of pathogenic afucosylated anti-spike IgGs. We found that FcγRIIa-transgenic mice injected with spike-expressing human MKs exhibited a significant increase in afucosylated anti-spike IgG production, platelet activity, and thrombosis rates. Importantly, inhibitors targeting either FcγRIIa, serotonin, or complement anaphylatoxins successfully prevent thrombus formation in these mice, revealing that these potential therapeutic targets might also reduce the risk of thrombosis in patients with severe forms of COVID-19.

## 2. Results

### 2.1. Proplatelets Derived from SARS-CoV-2-Infected Human MKs Carry Spike Protein on Their Membrane During Their Maturation

**Figure 1:**
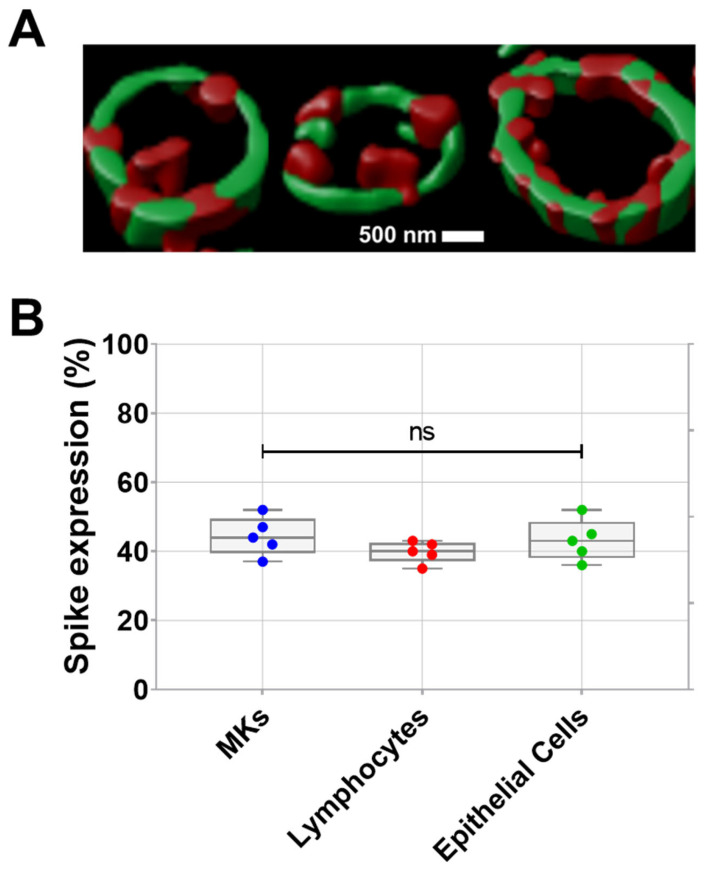
(**A**) Visualization of spike protein expression on pro-platelet surfaces using confocal microscopy following proplatelet fixation. Representative confocal microscopy image double stained showing α-tubulin that marks the periphery of pro-platelets from MKs (in green), and SARS-CoV-2 spike protein localized on the surface of pro-platelets (in red) taken at 63× magnification. (**B**) Quantification of spike expression (%), using flow cytometry, on MKs, lymphocytes, and epithelial cells from patients with COVID-19 (*n* = 5 patients). Data presented as min–max. Statistical comparisons were conducted using one-way ANOVA. ns = not significant.

Prior studies have revealed the infection of MKs by SARS-CoV-2 in the bone marrow of severe COVID-19 patients. Infected MKs express spike protein on their surface and may produce platelets containing SARS-CoV-2 [2,3]. Here, the expression of spike protein on the surface of proplatelets generated in vitro from human bone marrow MKs infected with SARS-CoV-2 was visually demonstrated by confocal microscopy (teaser) after proplatelet fixation. As shown in Figure 1A, the periphery of proplatelets appears in green as labeled with anti-α-tubulin, while SARS-CoV-2 spike protein appears in red on the proplatelets’ membrane. Spike expression in MKs from patients with COVID-19 further validated the presence of spikes on the surface of these SARS-CoV-2-infected MKs, in addition to SARS-CoV-2-infected lymphocytes and epithelial cells (Figure 1B).

### 2.2. SARS-CoV-2-Infected Human MK Injection Triggers the Formation of Afucosylated Anti-Spike IgGs and Promotes Platelet Activation, Lung Injury, and Death in FcγRIIa-Transgenic Mice

Anti-spike IgG antibodies with low level of Fc core fucosylation have been found to cause platelet hyperactivation, thrombosis and tissue damage [5,13,14], and to be associated with severe COVID-19 [4,5,6,7]. However, the factors triggering their formation during SARS-CoV-2 infection remain unclear. It has been shown that afucosylated IgG antibodies are not produced in response to soluble spike proteins in plasma, internal proteins of enveloped viruses, or proteins of non-enveloped viruses, suggesting that afucosylated anti-spike IgG responses are triggered by spike protein expressed on the membrane of infected host cells [4]. Since the spike protein is found expressed on the membrane of various SARS-CoV-2-infected host cells, such as MKs and their proplatelets, as shown in Figure 1, we asked whether a specific cell population expressing spike protein is responsible for the formation of these afucosylated antibodies that drive disease progression. On the other hand, this type of pathogenic antibodies also occurs against alloantigens expressed on the platelet’s membrane leading to FNAIT development [8,9]. Therefore, considering that MKs and their derived proplatelets and platelets are a pivotal element driving the generation of afucosylated antibodies against an antigen expressed on their membrane, we hypothesized that spike-expressing MKs and their proplatelets may trigger the production of afucosylated IgG responses in severe COVID-19 patients in a manner similar to that obtained by alloantigen-expressing platelets in FNAIT patients. To confirm this hypothesis, we investigated the level of afucosylated IgG antibodies produced against the spike protein in three distinct groups of FcγRIIa-transgenic mice injected with either MKs, epithelial cells, or lymphocytes expressing spike protein on their surface, all isolated from our cohort of COVID-19 patients.

**Figure 2:**
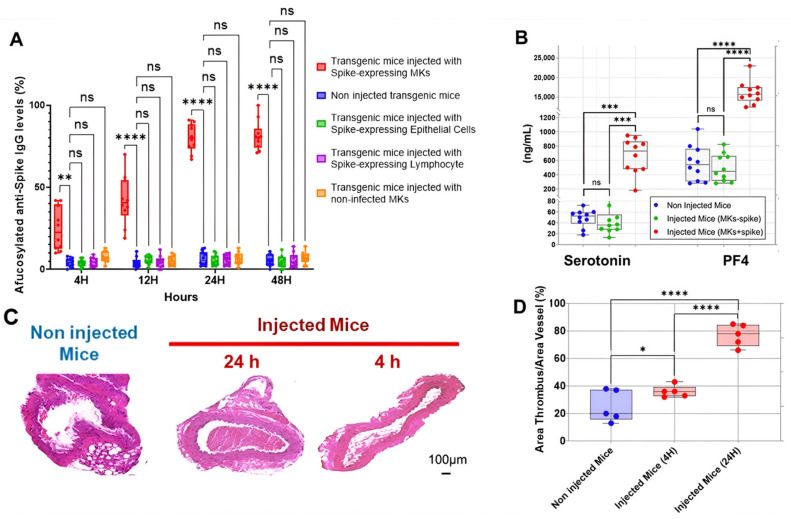
Spike-expressing human MK injection increases afucosylated anti-spike IgG, serotonin and PF4 levels, and causes pulmonary thromboembolism in FcγRIIa-transgenic mice. (**A**) The titers of afucosylated IgG antibodies against SARS-CoV-2 spike protein (%) were measured at 4 h, 12 h, 24 h, and 48 h following the second administration of spike-expressing cells or non-infected MKs to mice. High levels of afucosylated anti-spike IgG antibodies were observed in FcγRIIa-transgenic mice injected with spike-expressing human MKs, compared to those injected with either spike-expressing human epithelial cells, spike-expressing human lymphocytes, or non-infected human MKs, and to non-injected ones (n = 10 per group). (**B**) Plasma levels (ng/mL) of serotonin and PF4 were significantly elevated in FcγRIIa-transgenic mice at 24 h following the second injection of spike-expressing human MKs (MKs + spike) compared to mice injected with non-infected MKs (MKs—spike) and non-injected mice (n= 10 per group). (**C**,**D**) Histological analysis of the lung tissue stained with H&E using light microscopy (20×) showing the development of pulmonary thromboembolism in FcγRIIa-transgenic mice at 4 h and 24 h following the second injection of spike-expressing human MKs (**C**), characterized by a significantly increased thrombus area (%) (**D**) in contrast to uninjected mice (n = 5 per group). Data presented as min–max. Statistical comparisons were conducted using two-way ANOVA for panels (**A**,**B**), and by one-way ANOVA followed by an appropriate test for comparison against a single group for panel (**D**). * *p* < 0.05; ** *p* < 0.01; *** *p* < 0.001; **** *p* < 0.0001; ns = not significant.

**Figure 3:**
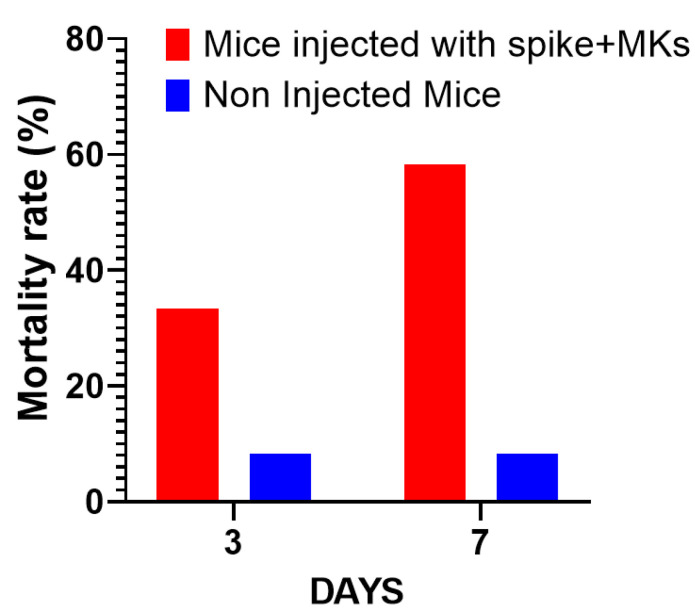
Comparison of mortality rates over time: Mice injected with spike-expressing MKs vs. non-injected mice. The graph shows the percentage of mortality in both groups across a span of days (*n* = 12 per group).

The first interesting result is shown in Figure 2A, indicating that the injection of human MKs infected by SARS-CoV-2 into FcγRIIa-transgenic mice significantly promoted the production of high-titered afucosylated IgG antibodies to the spike protein, in contrast to uninjected mice and mice injected with either spike-expressing human epithelial cells, spike-expressing human lymphocytes, or non-infected human MKs, which had low levels of afucosylated anti-spike IgG antibodies. Subsequently, we evaluated the effect of spike-expressing human MK injection on platelet activity in FcγRIIa-transgenic mice. Indeed, the majority of mouse strains do not naturally express the human ACE2 receptor and are, therefore, generally not susceptible to SARS-CoV-2 infection and the resultant disease manifestations [15,16,17]. This allowed us to specifically examine the effects of antibodies produced against the spike protein on platelet activation. As a result, the plasma levels of serotonin and PF4 were significantly increased in mice with spike-expressing MKs compared to those injected with non-infected MKs and uninjected ones (Figure 2B), suggesting that platelets were remarkably more activated in mice injected with spike-expressing MKs than in mice injected with intact MKs. This was confirmed by histological examination of lung tissue stained with Hematoxylin and Eosin using light microscopy (20×), which showed that the non-injected mice had a normal vessel and lung tissue, whereas in the other cross-sections at 4 h and 24 h post-second injection of the transgenic mice with human spike-expressing MKs, invading inflammatory cells and thrombus in the vessel lumen and distortions of the lung tissue can be seen (Figure 2C,D). We also noted that the survival rate was significantly lower in the injected group, with 33.3% mortality at day 3 and 58.3% at day 7, compared to only 8.3% mortality in the non-injected group throughout the study (Figure 3).

Taken together, these findings suggest that the incorporation of SARS-CoV-2 spike protein into platelets during their maturation may play a critical role in the pathogenesis of COVID-19 by promoting the formation of afucosylated IgG antibodies, which can lead to more severe disease outcomes, including thromboembolic diseases.

### 2.3. Inhibition of FcγRIIa Receptor, Serotonin Transporter, or Complement Anaphylatoxins Prevents Thrombosis by Reducing Platelet Activation and Aggregation in Injected FcγRIIa-Transgenic Mice with High Level of Afucosylated Anti-Spike IgGs

Human platelets express FcγRIIa receptor for IgG and serotonin transporter (SERT) on their membrane [11,18]. As we observed that transgenic mice injected with spike-expressing human MKs had high levels of afucosylated IgG antibodies and serotonin (Figure 2A,B), with a notable increase in thrombosis rate (Figure 2C,D), it is possible that these afucosylated IgG immune complexes bind with high affinity to platelet FcγRIIa, leading to an enhanced activation of platelets, which, in turn, release a high level of platelet activators, including serotonin. This dual action (of afucosylated IgG antibodies and serotonin) increases platelet activation, thereby resulting in thrombus formation. Therefore, we asked whether the treatment of these injected mice with inhibitors targeting FcγRIIa and SERT on the platelets’ surface would reduce platelet activation, aggregation, and thrombus formation. Since mouse platelets do not express the FcγRIIa receptor, we used a mouse model transgenic for the human FcγRIIa receptor (CD32a) [19,20] to evaluate the effect of platelet FcγRIIa inhibition in preventing thrombosis induced by afucosylated platelet-activating anti-spike IgG antibodies. Furthermore, IgG immune complexes trigger the activation of complement, generating the active and soluble anaphylatoxins C5a, and C3a, which also may contribute to platelet hyperactivity and thrombosis in patients with COVID-19 [21,22,23]. For this reason, the effects of inhibitors targeting C5a and C3a in mice with spike-expressing MKs were also investigated.

**Figure 4:**
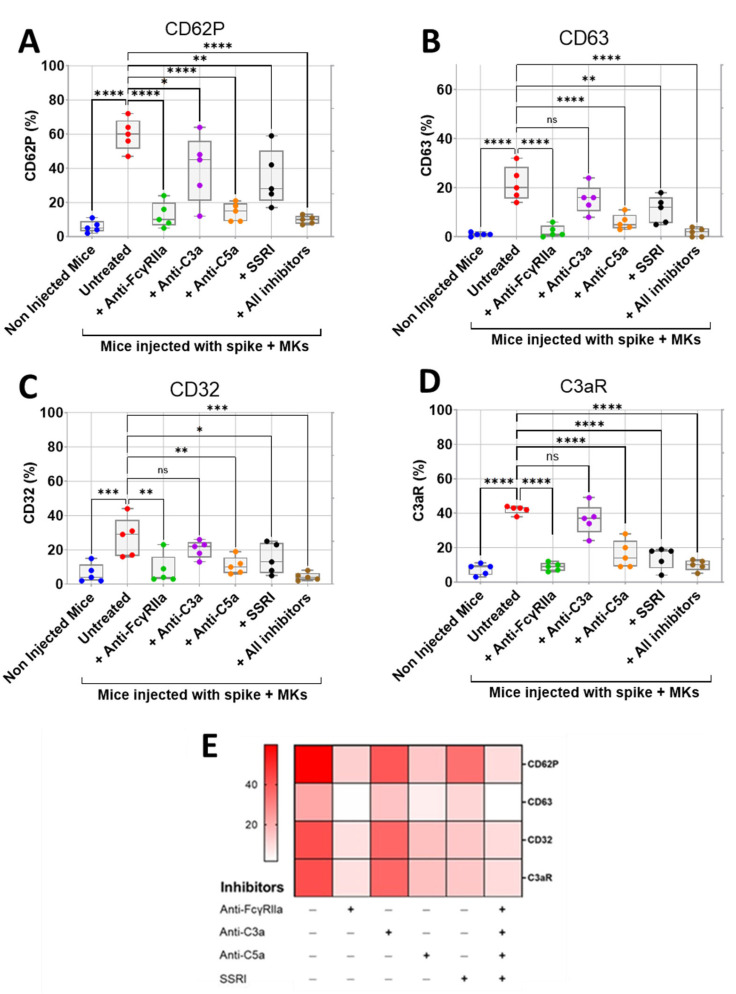
Inhibitory effects of FcγRIIa, SERT, and complement anaphylatoxin blockade on activation of platelets from FcγRIIa-transgenic mice injected with spike-expressing human MKs. At 24 h following the second injection of spike-expressing MKs, mice were injected into the jugular vein with either anti-FcγRIIa (5 mg/Kg), anti-C3a (10 mg/Kg), anti-C5a (10 mg/Kg) mAbs, SSRI (Fluvoxamine, 10 mg/Kg), or the combination of all four inhibitors for 5 min. Platelets were isolated from non-injected mice and injected ones untreated or pretreated with the inhibitors tested, and then platelet surface expression of CD62P, CD63, CD32, and C3aR was measured by flow cytometry and expressed as percentage platelet expression (%) (*n* = 5 per group). (**A**–**D**) Boxplots represent platelet surface expression of (**A**) CD62P, (**B**) CD63, (**C**) CD32, and (**D**) C3aR in injected mice treated with either anti-FcγRIIa, anti-C3a, anti-C5a mAbs, SSRI, or the combination of all four inhibitors in comparison to injected untreated mice and non-injected ones. (**E**) Heatmap showing a marked decrease in surface expression of CD62P, CD63, CD32, and C3aR on platelets from injected mice treated with either anti-FcγRIIa, anti-C5a mAbs, SSRI, or the combination of all four inhibitors, whereas this reduction was minimal in the presence of anti-C3a mAb. Dark color indicates high marker expression, and light color indicates low marker expression. Data presented as min–max. Statistical comparisons were conducted using one-way ANOVA, followed by an appropriate test for comparison against a single group. * p < 0.05; ** p < 0.01; *** p < 0.001; **** p < 0.0001; ns = not significant.

We first assessed the effect of inhibition of FcγRIIa, SERT, C5a, and C3a on the surface expression of activation biomarkers CD62P (P-selectin) and CD63 on platelets isolated from FcγRIIa-transgenic mice injected with spike-expressing human MKs using flow cytometry. We also investigated the effects of these inhibitions on platelet surface expression of CD32 (FcγRII) and C3aR, given their potential role in platelet activation mediated by immune complexes and/or complement anaphylatoxins. We observed that platelets from injected mice had significantly increased expression of CD62P, CD63, CD32, and C3aR, indicating hyperactivity compared to platelets from non-injected mice (Figure 4A–E). Interestingly, the treatment of injected mice with either anti-FcγRIIa, anti-C5a monoclonal antibodies (mAbs), SSRI (selective serotonin reuptake inhibitor fluvoxamine), or the combination of all four inhibitors (including anti-C3a mAb) resulted in a significant reduction in platelet surface expression of CD62P, CD63, CD32, and C3aR relative to untreated injected mice (Figure 4A–E). Comparatively, blocking FcγRIIa had the strongest inhibitory effect on platelet activation, followed by C5a neutralization, and then SERT inhibition. We also observed that the combination of all four inhibitors tested reduced platelet activation in a manner similar to that achieved by FcγRIIa inhibition (Figure 4A–E). In contrast, the treatment of injected mice with anti-C3a mAb caused a minimal reduction in platelet surface expression of these activation biomarkers, compared to the other three inhibitors tested (Figure 4A–E), and we observed no statistically significant difference in the expression of CD63, CD32, and C3aR between platelets from anti-C3a-treated and untreated injected mice, indicating that the inhibition of C3a has a weak inhibitory effect on platelet activation (Figure 4B–D).

**Figure 5:**
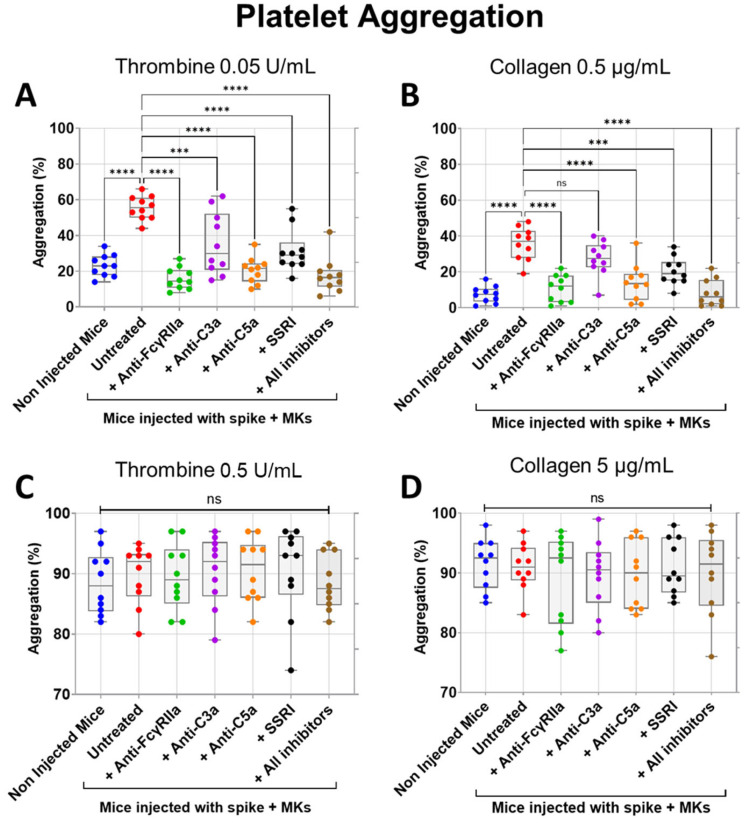
Inhibitory effects of FcγRIIa, SERT, and complement anaphylatoxin blockade on aggregation of platelets from FcγRIIa-transgenic mice injected with spike-expressing human MKs. At 24 h following the second injection of spike-expressing MKs, mice were injected into the jugular vein with either anti-FcγRIIa (5 mg/Kg), anti-C5a (10 mg/Kg), anti-C3a (10 mg/Kg) mAbs, SSRI (Fluvoxamine, 10 mg/Kg), or the combination of all four inhibitors for 5 min. Platelets were isolated from non-injected mice and injected ones untreated or pretreated with the inhibitors tested, were stimulated with different agonists, including (**A**) 0.05 U/mL thrombin, (**B**) 0.5 µg/mL collagen, (**C**) 0.5 U/mL thrombin, or (**D**) 5 µg/mL collagen, and then platelet aggregation was measured using an optical aggregometer (*n* = 10 mice per group). (**A**) Pretreatment of injected mice with anti-FcγRIIa, anti-C5a, anti-C3a mAbs, SSRI, or the combination of all four inhibitors significantly reduced platelet aggregation (%) induced by low-dose thrombin (0.05 U/mL). (**B**) Low-dose collagen (0.5 µg/mL)-induced aggregation of platelets (%) from injected mice was significantly reduced by pretreatment with anti-FcγRIIa, anti-C5a mAbs, SSRI, or the combination of all four inhibitors but was not significantly inhibited by pretreatment with anti-C3a mAb. (**C**,**D**) There was no significant difference in platelet aggregation (%) induced by (**C**) high-dose thrombin (0.5 U/mL) or by (**D**) high-dose collagen (5 µg/mL) between platelets from uninjected mice, untreated, and pretreated injected mice. Data presented as min–max. Statistical comparisons were conducted using one-wayANOVA, followed by an appropriate test for comparison against a single group. *** p < 0.001; **** p < 0.0001; ns = not significant.

We next tested the inhibitory effects of FcγRIIa, SERT, C5a, and C3a blockade on both thrombin- and collagen-induced aggregation of platelets isolated from FcγRIIa-transgenic mice injected with spike-expressing human MKs. As expected, platelets from injected mice were characterized by a marked increase in aggregation in response to low dose of thrombin (0.05 U/mL) or collagen (0.5 µg/mL) agonists in comparison with platelets from uninjected mice (Figure 5A,B). Notably, pretreatment of injected mice with anti-FcγRIIa, anti-C5a, anti-C3a mAbs, SSRI (fluvoxamine), or the combination of all four inhibitors significantly reduced low-dose thrombin (0.05 U/mL)-induced platelet aggregation, in contrast to injected untreated mice (Figure 5A). By comparison, FcγRIIa blockade exhibited the most potent inhibitory effect followed by C5a neutralization, while blocking SERT and C3a had a relatively weaker inhibitory effect on aggregation induced by thrombin 0.05 U/mL, compared to FcγRIIa and C5a inhibition. We also observed that the combination of all four inhibitors tested has an inhibitory effect almost similar to that obtained by C5a neutralization (Figure 5A). Likewise, injected mice pretreated with anti-FcγRIIa, anti-C5a mAbs, SSRI (fluvoxamine), or the combination of all four inhibitors (including anti-C3a mAb) also showed a significant reduction in platelet aggregation induced by low-dose collagen (0.5 µg/mL), where blocking FcγRIIa alone or all four pathways simultaneously had a robust inhibitory effect, followed by C5a neutralization, and then SERT inhibition, whereas anti-C3a mAb had no significant inhibitory effects on collagen (0.5 µg/mL)-induced platelet aggregation, as compared with untreated injected mice (Figure 5B). Furthermore, when platelets were stimulated with a high dose of thrombin (0.5 U/mL) or collagen (5 µg/mL), aggregation had reached its maximum level, and there was no significant difference observed between platelets from injected mice (either untreated or pretreated with tested inhibitors) and non-injected mice (Figure 5C,D).

**Figure 6:**
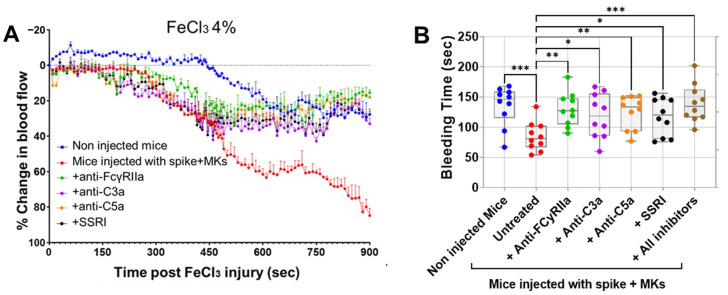
Treatment targeting either FcγRIIa, complement anaphylatoxins, or serotonin transporter reduces thrombosis rates in FcγRIIa-transgenic mice injected with spike-expressing human MKs. (**A**) At 24 h following the second injection of spike-expressing MKs, mice were injected into the jugular vein with either anti-FcγRIIa (5 mg/kg), anti-C5a (10 mg/kg), anti-C3a (10 mg/kg) mAbs, or SSRI (Fluvoxamine; 10 mg/kg), 5 min before carotid artery injury induced by 4% FeCl_3_, and thrombus formation was monitored by continuously measuring blood flow and assessing thrombus growth dynamics for 900 s post-FeCl_3_ injury and was expressed as %thrombosis (*n* = 3 per group). A significant reduction in thrombosis rates was observed in injected treated mice. (**B**) Tail bleeding time (in seconds) was measured in mice (at 24 h following the second injection of spike-expressing MKs) injected with either anti-FcγRIIa (5 mg/kg), anti-C5a (10 mg/kg), anti-C3a (10 mg/kg) mAbs, SSRI (Fluvoxamine; 10 mg/kg), or the combination of all four inhibitors for 5 min, injected untreated mice, and non-injected ones (*n* = 10 per group). A significant prolongation of bleeding time was observed in injected treated mice. Data presented as mean ± SEM for panel (**A**) and as min–max for panel (**B**). Statistical comparisons were conducted using one-way ANOVA, followed by an appropriate test for comparison against a single group. * p < 0.05; ** p < 0.01; *** p < 0.001.

Having shown that inhibition of FcγRIIa, SERT, or complement anaphylatoxins reduced activation and aggregation of platelets from FcγRIIa-transgenic mice injected with spike-expressing human MKs, we further examined whether they also prevent thrombosis using the FeCl_3_-induced arterial injury model. As seen in Figure 6A, mice injected with spike-expressing human MKs exhibited a marked increase in thrombosis rates following FeCl_3_-induced injury of the carotid artery, when compared with non-injected mice. This confirms our results presented in Figure 2C, where pulmonary thromboembolism in injected mice was visually observed. More importantly, the pretreatment of injected mice with either anti-FcγRIIa, anti-C5a, anti-C3a mAbs or SSRI (fluvoxamine) significantly reduced thrombosis following FeCl_3_-induced carotid artery injury, as compared with injected untreated mice (Figure 6A). For more investigation, the bleeding time was also measured using transection tail method. Compared with uninjected mice, mice injected with spike-expressing human MKs exhibited a significant reduction in bleeding time after tail transection. We observed that tail-bleeding time in injected mice pretreated with either anti-FcγRIIa, anti-C5a, anti-C3a mAbs, SSRI (fluvoxamine), or the combination of all four inhibitors was relatively longer than that in injected untreated mice (Figure 6B), confirming the results shown in Figure 6A. In terms of effectiveness, blocking FcγRIIa had the strongest antithrombotic effect, followed by C5a neutralization, then SERT inhibition, and finally, C3a neutralization in mice injected with spike-expressing MKs (Figure 6A,B). Of note, although the inhibition of C3a showed a modest inhibitory effect on platelet activation and aggregation in vitro (Figure 4A–E and Figure 5B), it effectively reduced thrombus formation in vivo, similar to the other three inhibitors tested (Figure 6A,B).

Collectively, our results demonstrate that the treatment with inhibitors targeting FcγRIIa, SERT, or complement anaphylatoxins successfully prevent thrombus formation in mice injected with spike-expressing human MKs, which were characterized by a high level of afucosylated anti-spike IgG antibodies, a marker of COVID-19 severity [4,5,6], thus revealing potential therapeutic targets that might prevent severe forms of the disease.

### 2.4. Afucosylated Anti-Spike IgG Levels Correlate with Pulmonary Thromboembolism, and Mortality in FcγRIIa-Transgenic Mice with Spike-Expressing Human MKs

**Figure 7:**
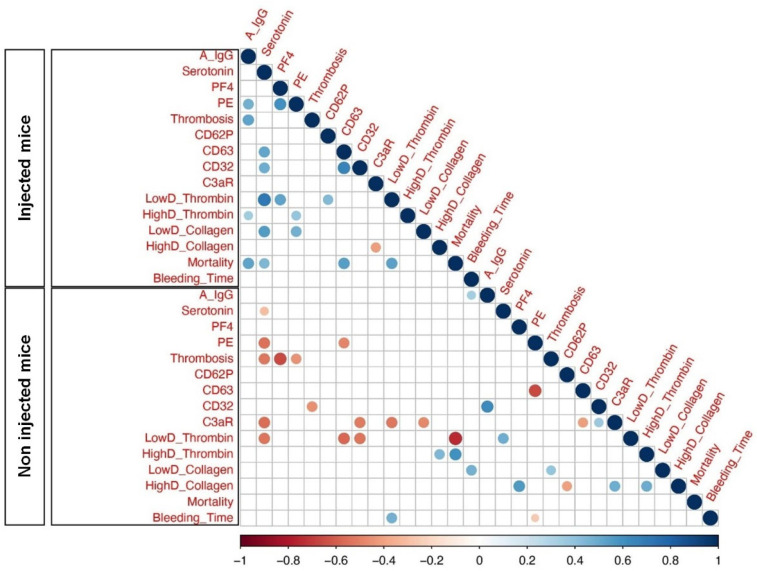
Heatmap of pairwise correlations between different parameters of Injected Mice and Non-injected Mice. Heatmaps represent afucosylated IgG levels at 24 h following the second administration of spike-expressing MKs: A IgG, Serotonin (ng/mL), PF4 (ng/mL), Pulmonary Embolisms (%): PE, Thrombosis: Thromb, CD62P, CD63, CD32, C3aR, Platelet Aggregation (Low and High Doses of Thrombin): LowD Thrombin and HighD Thrombin, Platelet Aggregation (Low and High Doses of Collagen): LowD Collagen and HighD Collagen, Mortality (%) and Bleeding Time (sec): BT. The size of the circles and different colors represent the correlation range (r) from −1 to +1. Red indicates a perfect negative correlation, whereas blue indicates a perfect positive correlation. The color key of the correlations is shown at the bottom. Light color indicates a low correlation, and dark color indicates a high correlation. Statistically significant (*p*-value less or equal to 0.05) correlations are shown with colored circles.

Considering that FcγRIIa-transgenic mice injected with spike-expressing human MKs exhibited a high level of afucosylated anti-spike IgG antibodies and were characterized by a significant increase in platelet activation, pulmonary thromboembolism, and mortality, we aimed to investigate the relationships between these variables using Spearman correlations. As a result, our analysis showed a significant positive correlation among afucosylated anti-spike IgGs and the incidence of thrombosis and pulmonary thromboembolism in mice injected with spike-expressing human MKs, as compared to non-injected ones. Moreover, afucosylated anti-spike IgGs was also positively correlated with the mortality rate in these injected mice. Furthermore, there was no significant positive correlation between the levels of afucosylated anti-spike IgGs and other parameters, such as platelet activation biomarkers (Figure 7).

Overall, our correlation analysis revealed a potential positive correlation between afucosylated IgG antibody levels against the spike protein, pulmonary thromboembolism, and mortality rate in mice injected with spike-expressing human MKs, indicating that these relationships may also be present in patients with severe COVID-19. While our findings are corroborated by a prior in vitro study that demonstrated an association between afucosylated anti-spike IgGs and increased platelet activation and thrombus formation [13], it is imperative that future in vivo studies, including the direct injection of isolated afucosylated anti-spike IgGs, be conducted to validate this relationship.

## 3. Discussion

**Figure 8:**
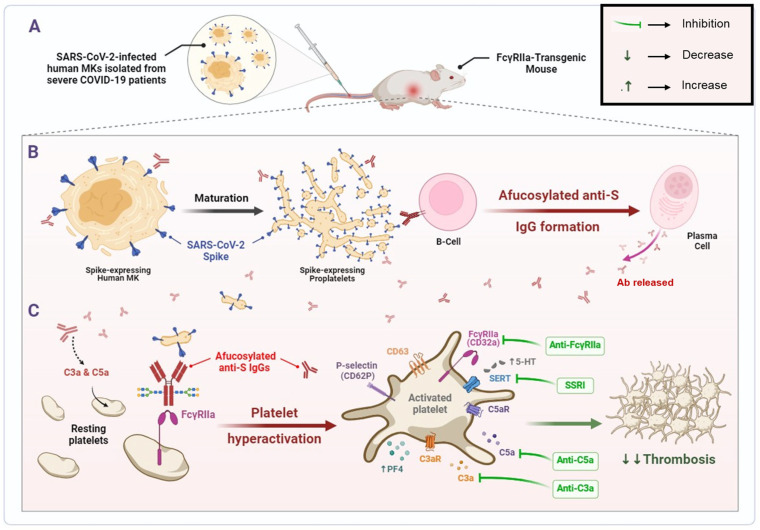
SARS-CoV-2-infected human MK injection mediates the formation of pathogenic afucosylated anti-spike IgGs, resulting in platelet hyperactivation and thrombosis in FcγRIIa-transgenic mice. (**A**) Bone marrow megakaryocytes (MKs) from severe COVID-19 patients are infected by SARS-CoV-2. (**B**) Pro-platelets derived from SARS-CoV-2-infected human MKs carry SARS-CoV-2 spike protein on their membrane during their maturation. Spike-expressing human MKs and their proplatelets promote the formation of pathogenic afucosylated anti-spike IgG antibodies in FcγRIIa-transgenic mice. (**C**) Afucosylated IgG immune complexes bind directly to their high affinity FcγRIIa receptor on platelet surface, which may mediate platelet hyperactivation (as represented by surface expression of CD62P and CD63), granule release (including serotonin and PF4) and may have caused thrombosis in FcγRIIa-transgenic mice injected with spike-expressing human MKs. Treatment with inhibitors targeting either FcγRIIa, SERT, or complement anaphylatoxins prevent thrombus formation in these mice. Afucosylated anti-S IgGs, Afucosylated anti-Spike Immunoglobulins G; C3a, third complement component-derived anaphylatoxin; C5a, fifth complement component-derived anaphylatoxin; C3aR, C3a receptor; C5aR, C5a receptor; MKs, megakaryocytes; PF4, platelet factor 4; SERT, serotonin reuptake transporter; SSRI, selective serotonin reuptake inhibitor; 5-HT, serotonin.

The findings of this study depict that human MKs infected with SARS-CoV-2 and expressing spike protein on their membrane are a pivotal element promoting the formation of pathogenic afucosylated anti-spike IgG antibodies associated with severe COVID-19. These infected MKs with afucosylated antibodies trigger enhanced platelet activation, particularly via FcγRIIa signaling, resulting in increased expression of platelet activation biomarkers, including CD62P, CD63, and the release of platelet activators, such as serotonin (5-HT) and PF4 (CXCL4). Thus, platelets become hyperactive leading to thrombus formation, pulmonary thromboembolism, and death. Subsequently, treatment with inhibitors targeting either FcγRIIa, serotonin transporter, or complement anaphylatoxins may prevent these complications. The mechanisms revealed in this study are illustrated in Figure 8.

So far, there is a lack of knowledge about the factors triggering the production of afucosylated IgG responses. It has been suggested that afucosylated IgG responses occur mainly against foreign antigens expressed on the membrane of host cells particularly host blood cells, such as platelets and red blood cells [4,8,9,24]. These antigens may be alloantigens or outer-membrane proteins of enveloped viruses [4,8,9,24,25]. In the case of severe COVID-19, higher titers of early afucosylated IgG antibodies specifically directed against SARS-CoV-2 spike protein have been reported [4,5,6,7]. However, in contrast to acute dengue virus infection [26], SARS-CoV-2 virus infection itself have not been shown to regulate antibody glycosylation, including fucosylation during infection [14]. A study indicates that this response was not triggered by soluble spike proteins, suggesting that it was restricted to spikes embedded in the membrane of infected host cells [4]. In line with this knowledge, we hypothesized that the production of anti-spike IgG antibodies with low level of Fc core fucosylation may be promoted by one of the cell populations infected by SARS-CoV-2, those which are able to express spike protein on their membrane, especially platelets, since they have been observed to be involved in the formation of afucosylated IgG antibodies against alloantigens expressed on their membrane in patients with FNAIT [8,9].

Previous reports have shown the presence of spike protein on the surface of bone marrow MKs and their derived platelets from patients with severe and fatal forms of COVID-19 [2,3]. These infected MKs are considered a primary source of platelets containing SARS-CoV-2, suggesting a potential role for this process in the immunothrombosis associated with severe COVID-19 [3]. Accordingly, in the current study, we demonstrated that these infected human bone marrow MKs are capable of generating proplatelets that carry the spike protein on their surface during platelet maturation in vitro, indicating that this phenomenon may also occur in vivo. If this were to happen in vivo, released spike-expressing proplatelets/platelets can be recognized by B cells and trigger the production of afucosylated anti-spike IgG antibodies, as it has been shown in FNAIT patients, whose afucosylated IgG responses occur against alloantigens expressed on platelet membrane [8,9]. This was confirmed in our study in which high levels of afucosylated anti-spike IgG antibodies were observed only in mice injected with spike-expressing human MKs but not in mice injected with either spike-expressing human epithelial cells or spike-expressing human lymphocytes. Although our analysis did not distinguish between IgG subclasses, previous studies have shown that afucosylation predominantly affects the IgG1 subclass, which is known for its high pro-inflammatory potential [4,6,7,8]. Future studies involving subclass-specific glycol-profiling will be necessary to precisely determine which IgG subclass mediates the observed effects.

Based on our investigation, it appears that the presence of spike protein on the membrane of pro-platelets and platelets derived from SARS-CoV-2-infected human MKs could also potentially lead to the production of pathogenic afucosylated IgG antibodies in patients with severe COVID-19 in a manner analogous to that seen in our experimental model. This phenomenon is likely to occur within the bone marrow and lungs, two critical sites of thrombopoiesis. The bone marrow is the primary site of megakaryopoiesis, where SARS-CoV-2 can infect MKs and promote spike presentation on emerging proplatelets [2]. In the pulmonary vasculature, circulating MKs can further mature and generate platelets, making the lungs a secondary thrombopoietic site. These two anatomical sites are thus central to spike exposure and subsequent afucosylated IgG production. The aberrant expression of spike protein on the pro-platelets’ membrane could potentially be recognized by the immune system as a highly abnormal signal, which could potentially trigger the formation of highly potent afucosylated IgG antibodies specifically targeting spike protein. Inter-individual differences in the generation of afucosylated IgG responses may arise from a combination of factors, including genetic or epigenetic variations influencing fucosyltransferase expression in B cells, pro-inflammatory cytokine milieus (e.g., elevated IL-6 or type I interferons), and the immunological context in which spike antigen is encountered, membrane-bound on infected MKs versus soluble in circulation [27,28,29]. These elements may synergize to create a pro-afucosylation environment in certain individuals, thereby increasing their susceptibility to severe disease. Our data may therefore assist in identifying potential mechanisms and pathways involved in the generation of afucosylated IgG responses during SARS-CoV-2 infection, highlighting the crucial role of spike-expressing MKs in this process. These afucosylated IgG antibodies are pathogenic and contribute significantly to the severity of COVID-19 [4,5,6,7].

In general, antibodies play a pivotal role in host defense against infections, using diverse mechanisms to counteract pathogens [30]. The effectiveness of these mechanisms is influenced by intrinsic characteristics of the antibodies, including isotype, subclass, allotype and glycosylation [31]. In the context of critically ill COVID-19 patients, alterations in the glycosylation patterns of anti-spike IgG antibodies have been observed [4,5,6,7]. These modifications amplify the effector functions of IgG, leading to heightened pro-inflammatory cytokine production [32,33]. Notably, decreased fucosylation of anti-spike IgG antibodies was shown in vitro to enhance affinity for FcγRIIa and FcγRIII receptors, promoting the production of high levels of inflammatory cytokines by monocytes/macrophages, including interleukin-6 and tumor necrosis factor, thus causing excessive inflammatory responses (cytokine storm) [5,14]. These findings suggest the involvement of anti-spike IgG antibodies, namely those lacking core fucosylation in the induction of pulmonary inflammation and tissue damage in severe COVID-19 cases [5].

In addition to the cytokine storm-induced pulmonary inflammation, severe forms of COVID-19 are characterized by the presence of pulmonary edema resulting from microvascular endothelial rupture [34], as well as coagulopathy leading to pulmonary immuno-thrombosis in many patients [35,36,37]. Afucosylated anti-spike IgG antibodies may also contribute to platelet hyperreactivity leading to thrombosis, either indirectly by activation of macrophages (via FcγRIIa and FcγRIII receptors) and disruption of pulmonary endothelium [14] or by other mechanisms, such as complement activation [7], or directly by binding to platelet FcγRIIa [13]. While some evidence indicates that afucosylated antibodies exhibit high-affinity binding for FcγRIII yet not for FcγRIIa [38], other findings suggest enhanced interactions with both Fc receptors [5,13]. Indeed, a previous study has shown that immune complexes containing SARS-CoV-2 spike protein and anti-spike IgGs with both low fucosylation and high galactosylation in their Fc region enhanced platelet-mediated thrombosis on von Willebrand factor through platelet FcγRIIa stimulation in vitro [13], further supporting our findings in FcγRIIa-transgenic mice injected with spike-expressing human MKs that are characterized by a high level of afucosylated anti-spike IgG antibodies. Future in vivo studies, including direct injection of isolated afucosylated anti-spike IgGs, will be essential to confirm this relationship and further explore the underlying mechanisms independent of other human cell-derived factors. Overall, the above data confirm the hypothesis that altered anti-spike IgG glycosylation is sufficient to lead to platelet hyperreactivity and thrombosis in a manner similar to that caused by circulating MKs themselves [3], direct binding of SARS-CoV-2 spike protein to platelet ACE2 [39], SARS-CoV-2 RNA [37], or the virus itself [40] in severe cases of COVID-19. These immune mechanisms involving afucosylated IgG antibodies may explain the activation of platelets and subsequent release of alpha and dense granule contents, such as PF4 and serotonin, as well as the development of pulmonary thromboembolism and increased mortality observed in FcγRIIa-transgenic mice injected with spike-expressing human MKs, which appear to be correlated with afucosylated anti-spike IgG levels produced in these mice. Therefore, treatment with inhibitors primarily targeting FcγRIIa signaling may prevent thrombosis induced by afucosylated platelet-activating anti-spike IgG antibodies.

Interestingly, we found that blocking FcγRIIa receptor with monoclonal antibody markedly reduced observed platelet hyperactivation and thrombosis induced by immune complexes containing afucosylated anti-spike IgG antibodies in FcγRIIa-transgenic mice injected with spike-expressing human MKs, revealing a strong antithrombotic effect in these mice. A previous report indicates that the inhibition of FcγRIIa-mediated platelet activation signaling, either through receptor blockade, IgG depletion or Syk inhibition, robustly reduced in vitro platelet hyperactivation induced by the plasma of COVID-19 patients [22]. Another in vitro study supporting our findings reported that the inhibition of FcγRIIa signaling, either through blocking FcγRIIa receptor, inhibition of Syk or inhibition of Btk, significantly reduced platelet activation and thrombosis induced by immune complexes containing anti-spike IgGs with both low fucosylation and high galactosylation in the Fc domain [13]. In addition to FcγRIIa inhibition, inhibition of serotonin reuptake transporter (SERT) with fluvoxamine, a selective serotonin reuptake inhibitor (SSRI) also exhibited a significant inhibitory effect by reducing platelet hyperactivation and thrombosis induced by immune complexes containing afucosylated anti-spike IgG antibodies in our experimental model. Indeed, higher levels of platelet serotonin are observed in the plasma of transgenic mice injected with spike-expressing human MKs, as previously reported in patients with severe COVID-19, indicating degranulation of platelet-dense granules as a result of platelet activation [41]. Early treatment of COVID-19 patients with SSRI agents has shown a beneficial effect by reducing the risk of developing severe symptoms and mortality [42,43]. Additionally, released serotonin is known to enhance platelet activation and aggregation by binding to 5-HT2 and 5-HT3 receptors on the platelet surface [44,45]. Thus, early treatment of COVID-19 patients with inhibitors targeting 5-HT2 and 5-HT3 receptors could potentially reduce the pathogenic effects of excessive serotonin levels, including thrombosis and thrombotic complications, potentially preventing progression to severe disease [42,44]. Therefore, it might be required to also investigate the inhibitory effects of 5-HT2 and 5-HT3 antagonists on thrombosis induced by afucosylated platelet-activating anti-spike IgG antibodies in mice injected with spike-expressing MKs, and to check whether they can provide the same beneficial effect as depletion of serotonin with SSRI use.

In addition to high levels of afucosylated anti-spike IgG antibodies, higher concentration of anaphylatoxins C3a and C5a has also been reported in the serum of patients with severe COVID-19 [23]. Recent research has shown that COVID-19 patients during acute COVID-19 and at 6-month follow-up are characterized by persistent dysregulation of the complement system with ongoing activation of the classical complement pathway by IgG antibodies and increased platelet activation markers, considering them as biomarkers of long COVID [46]. Although no differences in serum anti-spike IgG levels were observed between these patients and those without 6-month-long COVID [46], it is possible that the glycosylation pattern of anti-spike IgG in long-COVID patients differs from the one in patients without 6-month-long COVID. As a hypothesis, immune complexes containing afucosylated anti-spike IgG antibodies could potentially enhance complement activation. Overactivation of the complement system may contribute to thrombotic events in COVID-19 patients through the generation of high levels of anaphylatoxins C3a and C5a, which bind to their respective receptors C3aR and C5aR on platelets, thereby resulting in platelet hyperactivation and thrombosis [47,48,49]. In this report, neutralization of C3a or C5a anaphylatoxins by monoclonal antibodies significantly decreased observed platelet hyperactivation and thrombosis induced by immune complexes containing afucosylated anti-spike IgG antibodies in mice injected with spike-expressing human MKs. Especially, inhibition of C5a had a greater antithrombotic effect than inhibition of C3a in these mice. A previous study consistent with our results showed that C5a neutralization significantly reduced in vitro platelet hyperactivation induced by the plasma of COVID-19 patients, while C3a inhibition had the least inhibitory effect [22]. More importantly, targeting the complement system through inhibition of complement components, such as C3, C5, C5a and C5aR, has been suggested as a potential therapeutic strategy to reduce hyperinflammation, risk of thrombosis and other complications associated with severe COVID-19 [21,48,50,51,52].

In summary, the present findings underscore the urgent need to further investigate and elucidate the signaling pathways that initiate the generation of afucosylated IgG antibodies during infection, with a particular focus on infected MKs as highly suspected contributors to this phenomenon. Additionally, further comparative studies involving other pathogens affecting MKs, such as dengue virus and human immunodeficiency virus (HIV), are needed to determine whether this mechanism is specific to SARS-CoV-2 or shared by other viruses affecting MK behavior. Such understanding would enable the development of tailored vaccines that can elicit the desired fucosylated Fc antibody response. Moreover, it would facilitate the identification of therapeutic strategies aimed at preventing the formation of these pathogenic antibodies, which play a significant role in the morbidity and mortality associated with COVID-19, dengue fever, malaria, HIV, and other diseases. Furthermore, our data in the mouse model indicate a potential pathogenic role of anti-spike IgG responses with low core fucosylation, wherein the heightened activation of platelets enhanced by afucosylated anti-spike IgG antibodies can lead to thrombosis, thereby exacerbating disease. It should be noted that although the present results strongly support a link between spike-expressing MKs and afucosylated IgG-mediated pathology, the use of whole human MKs introduces potential confounders. Further in vivo experimental validation using purified afucosylated IgG antibodies is therefore necessary to determine their precise role in thrombosis induction and to confirm the current findings. Finally, we identified potential therapeutic targets to reduce the induced platelet hyperactivation and thrombosis, which may prevent thrombotic events in critically ill COVID-19 patients. While our data clearly demonstrated the antithrombotic effects of the tested inhibitors, the study did not extend to long-term survival analysis. Future experiments are warranted to determine whether these inhibitors confer a survival benefit in models of SARS-CoV-2-induced thrombosis.

## 4. Materials and Methods

### 4.1. Study Design

After obtaining informed consent and with the approval of the local ethics committee (No. PR-1113–22), MKs, lymphocytes, and epithelial cells were isolated from a cohort of human subjects infected by SARS-CoV-2 who were experiencing severe COVID-19 (*n*= 5 patients). The isolated cells were transferred to an appropriate culture medium containing growth factors and allowed to develop and differentiate under favorable growth conditions. The presence of SARS-CoV-2 was confirmed in the isolated cells using real-time PCR. Synthetic RNA for 2019-nCoV *E* gene and *RdRp* assay were used as positive controls (ACAGGTACGTTAATAGTTAATAGCGT and GTGARATGGTCATGTGTGGCGG for *E* and *RdRp* genes, respectively). For epithelial cells and lymphocytes, the RT-PCR assay was conducted immediately following cell isolation, while for MKs, the RT-PCR assay was conducted immediately upon completion of the 14-day culture period of MKs. Isolated cells confirmed to harbor SARS-CoV-2 were then injected intravenously into FcγRIIa-transgenic mice and a second injection was administered one week later at the same dose. Mice were monitored for signs of infection, disease, or behavioral changes. Blood samples were collected to assess the response to injection of the mice with virus-infected human cells, and to quantify the levels of afucosylated anti-spike IgG antibodies and other molecules (serotonin and Platelet Factor 4 (PF4)). Pharmacological inhibitors targeting FcγRIIa receptor, serotonin transporter, and complement anaphylatoxins were used to evaluate their inhibitory effects on platelet activation, aggregation, and thrombosis in mice injected with spike-expressing human MKs. Our study examined male and female mice, and similar findings are reported for both sexes.

### 4.2. Human MK Isolation

Briefly, bone marrow samples were collected into K3EDTA tubes from routine bone marrow biopsies that were obtained from our cohort of patients with severe COVID-19 and healthy donors (*n* = 5 per groups), and were diluted in a 1:1 ratio with DPBS (supplemented with 2% FBS) and deposited on the Lympholyte-H (1.5 mL) followed by centrifugation at room temperature for 30 min at 800× *g* and the small band of cells was obtained. The obtained cells were transferred to a new 5 mL tube and were centrifuged with 2–3 mL of DPBS at 300× *g* for 5 min. This step was repeated a second time and then the pellet was resuspended in 1 mL of fully supplemented IMDM followed by cell count. Cell suspension was cultured with 5 mL of fully supplemented IMDM with cytokines (TPO to the final concentration of 50 ng/mL and SCF to a final concentration of 20 ng/mL) and was incubated in 37 °C, 5% CO_2_ for 14 days by regularly changing the media every 3 days. During these steps, MK differentiation and maturation were assessed using flow cytometry after double staining with FITC-conjugated mouse anti-human CD41 (HIP8) and APC-conjugated mouse anti-human CD42b (HIP1) antibodies (BioLegend, San Diego, CA, USA) on days 0, 7, and 14, ensuring adequate monitoring of the culture to detect any potential defects in megakaryopoiesis. After 14 days, mature MKs were separated with BSA gradient after centrifugation at 200× *g* for 5 min and were cultured with 3 mL of fully supplemented IMDM in tissue-culture treated culture vessels for 24–48 h. Mature MKs produced proplatelets after 24–48 h of cell culture [53].

### 4.3. Confocal Microscopy

MK-derived pro-platelets were fixed with paraformaldehyde and allowed to immobilize on poly-L-lysine-coated coverslips. Adhered pro-platelets were permeabilized with Triton X-100. Pro-platelets were then incubated with the human monoclonal anti-SARS-CoV-2 Spike S1 antibody (Antikörper online, Aachen, Germany) and the mouse anti-human monoclonal antibody against α-tubulin (Santa Cruz Biotechnology, Dallas, TX, USA), washed and labeled with rabbit anti-human IgG–Alexa 555 (A001337128STJ) and anti-mouse IgG Alexa-488 secondary antibodies. A series of fluorescent confocal images (teasers) were acquired with a LSM-510 confocal microscope (Zeiss, Oberkochen, Germany).

### 4.4. Preparation of Cells for Injection

Cell suspensions for injection were prepared from samples collected from our cohort of severe COVID-19 patients (*n* = 5). MKs were purified from bone marrow samples as described above. For lymphocyte preparation, the peripheral blood mononuclear cells (PBMCs) were purified from peripheral whole blood samples by Ficoll density gradient centrifugation [54]. Then, lymphocytes were purified from isolated PBMCs by negative selection using CD14 immunomagnetic microbeads according to the manufacturer’s instructions (Miltenyi Biotec, Bergisch Gladbach, Germany). Nasal epithelial cells were collected by brushing of the inferior turbinate with a sterile brush as previously described [55]. The presence of SARS-CoV-2 was confirmed in the isolated MKs, lymphocytes, and epithelial cells using real-time PCR. The prepared cell suspensions (MKs, lymphocytes, and epithelial cells) were adjusted at a concentration of 5 × 10^5^ cells/mL in PBS. Each cell suspension was injected intravenously into the tail vein of a group of healthy mice (5 mL/kg) for immunization, and then a second injection was administered one week later at the same dose.

### 4.5. Preparation of Murine Platelets

FcγRIIa-transgenic mice used in this study were obtained from The Jackson Laboratory. Mice were bred and housed under pathogen free conditions. Handling and care of mice were in compliance with the guidelines established by the animal care and ethical committee of the Faculty of Sciences (CEFSR/APR/2023-APR02). Blood was collected by ventricular puncture from mice (2–3 months; 18–31 g) anesthetized with a mixture of 75 mg kg^−1^ of Ketamine (Vetalar, Zoetis, Kalamazoo, MI, USA) and 0.5 mg kg^−1^ of Medetomidine (Domitor, Pfizer, New York, NY, USA). Washed platelets were prepared from PRP and resuspended in modified Tyrode buffer to a final concentration of 250 × 10^6^/mL [56].

### 4.6. Flow Cytometry Analysis

For spike protein expression analysis, isolated MKs, lymphocytes, and epithelial cells were stained with FITC-conjugated mouse anti-human CD41 (clone HIP8), PE-conjugated mouse anti-human CD3 (clone OKT3) and APC-conjugated mouse anti-human CD326 (EpCAM) (clone 9C4) antibodies (BioLegend), respectively. Subsequently, each cell population was labeled with an anti-SARS-CoV S-D3 fragment antibody (clone 7G12, Merck Millipore, Burlington, MA, USA), followed by flow cytometry analysis.

For platelet surface markers, surface expression of CD62P, CD63, CD32, and C3aR on platelets were measured by flow cytometry, as previously described [56]. Mice injected with spike-expressing MKs were injected into the jugular vein with either anti-FcγRIIa (RayBiotech Life, Peachtree Corners, GA, USA; 5 mg/kg), anti-C5a (PA5-47260) (Invitrogen Corporation, Carlsbad, CA, USA; 10 mg/kg), anti-C3a (HM1072) (HycultBiotech, Wayne, PA, USA; 10 mg/kg) mAbs, SSRI Fluvoxamine (FLUVOX25; 10 mg/kg), or the combination of all 4 inhibitors for 5 min. Next, platelets were isolated from injected mice pretreated with the inhibitors tested or not and from non-injected ones, fixed with 1% paraformaldehyde, washed, and stained with saturating concentrations of the appropriate antibodies: BB700-conjugated rat anti-mouse CD62P (BD Biosciences, Franklin Lakes, NJ, USA, clone RB40.34), PE-conjugated rat anti-mouse CD63 (BioLegend, clone NVG-2), FITC-conjugated mouse anti-human CD32 (BD Biosciences, clone FLI8.26), and BV480-conjugated rat anti-mouse C3aR (BD Biosciences, clone 14D4). Samples were analyzed (20,000 events) on an Altra flow cytometer (Beckman Coulter, Mississauga, ON, USA) and platelets were gated by their characteristic forward and side scatter properties.

### 4.7. Platelet Aggregation Assay

Aggregation of washed platelets was monitored on an eight-channel optical aggregometer (SD Medical Innovation, Frouard, France). Platelets were isolated from mice injected with spike-expressing MKs and pretreated with appropriate inhibitors for 5 min (as described above) or not and from non-injected ones. Samples (500 μL of the isolated platelet suspension) were stimulated with collagen (0.5 and 5 µg/mL) (Chronolog Corp. Havertown, PA, USA) or thrombin (0.05 and 0.5 U/mL) (Sigma Aldrich, St. Louis, MO, USA), under continuous stirring (1000 rpm) at 37 °C. Platelet aggregation was then recorded until trace stabilization (or a maximum of 15 min) and light transmission was measured at the time of maximum aggregation.

### 4.8. Purification of Total IgG Antibodies

Affinity purification of total IgGs from sera was performed as previously described [57]. In brief, serum samples were collected from three distinct groups of FcγRIIa-transgenic mice immunized with either MKs, epithelial cells, or lymphocytes expressing spike protein on their surface and were diluted in PBS. IgG antibodies were purified from 15 µL of diluted serum samples by using Protein G-Sepharose 4 Fast Flow beads (GE Healthcare, Uppsala, Sweden) in a 96-well filter plate (Millipore Multiscreen, Amsterdam, The Netherlands). Captured IgG molecules were washed with PBS and LC-MS pure water, eluted with 100 µL of 100 mM formic acid, and then dried in a vacuum at room temperature.

### 4.9. Mass Spectrometry for Afucosylated Anti-Spike IgG Quantification

Quantitative analysis of mouse IgG Fc-specific glycan was performed by mass spectrometry as described before [58,59]. Briefly, IgGs were isolated from serum as described above. Spike-specific IgGs were isolated on agarose resin coupled to the spike protein followed by tryptic digestion of purified IgG bound to antigen-coated beads [14]. Subsequently, a nano-reverse phase liquid chromatography system coupled to mass spectrometry (nano-LC-ESI-MS) analysis was performed to determine the level of fucosylation at Asn-297 glycan site as previously described [58,59]. High-throughput processing of LC-MS data was carried out using LaCyTools software version 1.0.1 [59], which enabled precise quantification of fucosylated and non-fucosylated glycan species.

### 4.10. PF4 and Serotonin Quantification in Plasma

Mouse blood was drawn from the inferior vena cava into tubes containing 0.1 volume of 3.8% citrate. Platelet-poor plasma was prepared by centrifuging whole blood collected at 4500× *g* for 15 min and stored at −80 °C until use. PF4 contained in plasma was measured using the mouse CXCL4/PF4 DuoSet ELISA (R&D systems, Minneapolis, MN, USA) according to the manufacturer’s protocol. Samples were diluted to fall within the detection range of the ELISA kit. The Serotonin ELISA Fast Track kit (LDN, Nordhorn, Germany) was used to determine serotonin concentration in plasma according to the manufacturer’s instructions.

### 4.11. Tail-Bleeding Time

Mice (2–3 months; 18–31 g) were anesthetized by intra-peritoneal injection of a mixture of 75 mg/kg Ketamine and 0.5 mg/kg Medetomidine. Subsequently, inhibitors were administered into the jugular vein (2 μL/g of animal weight) at an adjusted concentration that eventually corresponds to the appropriated concentration 5 min prior to tail transection. Mice tails were transected at 3 mm from the tip using a scalpel blade and then immediately immersed in 0.9% isotonic saline at 37 °C. Bleeding time was defined as the duration necessary for the cessation of blood flow.

### 4.12. Pulmonary Thrombo-Embolism

Following the tail-bleeding time experiments, mice lungs were excised, fixed in 10% buffered formalin, embedded in paraffin, sectioned, stained with hematoxylin and eosin (H&E) and analyzed for the presence of thrombo-emboli within the pulmonary vasculatures [60]. Samples were visualized using an Olympus BX60 microscope (Olympus imaging America Inc., Center Valley, PA, USA) and images were captured with a Retiga 2000R camera (QImaging Corporation, Surrey, BC, USA), visualized and analyzed by the Image Pro Plus 7.0 software (Media Cybernetics, Rockville, MD, USA).

### 4.13. FeCl_3_-Induced Arterial Thrombosis

The effect of MK injection on thrombus formation was determined as previously described [56]. Briefly, anesthetized mice were injected with inhibitors anti-Mouse Fc gamma RIIa (RayBiotech Life, Peachtree Corners, GA, USA), 5 mg/kg, complement C5a Antibody (PA5-47260) (Invitrogen Corporation, Carlsbad, CA, USA), 10 mg/kg, complement C3a Antibody (HM1072) (HycultBiotech, Wayne, PA, USA), 10 mg/kg, or Fluvoxamine (FLUVOX25) 10 mg/kg through the jugular vein, 5 min prior to FeCl_3_ (4%) injury of the right carotid artery, and blood flow as well as thrombus development (blood flow of 0 mL/minute) were monitored with the aid of a miniature ultrasound flow probe (0.5 VB 552, Transonic Systems, Ithaca, NY, USA) interfaced with a flow meter (T206, Transonic Systems) and a computer-based data acquisition program (Iox 2.2.17.19, Emka, Falls Church, VA, USA).

### 4.14. Statistical Analysis

Results are presented as the mean ± SEM. Statistical comparisons were conducted using one-way ANOVA, followed by an appropriate test for comparison against a single group, two-way ANOVA, or unpaired Chi-square test. Data with *p* ≤ 0.05 were considered statistically significant. Statistical analyses and graphs were made using GraphPad Prism version 9.5.0. Spearman’s rank correlation coefficients were calculated to assess associations between variables. Correlation matrices were visualized using a correlogram, generated with the corrplot package in R version 4.3.3. Statistically significant (*p*-value less or equal to 0.05).

## Data Availability

Any data that support the findings of this study are available from the corresponding author upon reasonable request.
